# Short- and Long-Term Dentin Bond Strength of Bioactive Glass-Modified Dental Adhesives

**DOI:** 10.3390/nano11081894

**Published:** 2021-07-23

**Authors:** Ramona Oltramare, Matej Par, Dirk Mohn, Daniel B. Wiedemeier, Thomas Attin, Tobias T. Tauböck

**Affiliations:** 1 Clinic of Conservative and Preventive Dentistry, Center for Dental Medicine, University of Zurich, 8032 Zurich, Switzerland; dirk.mohn@chem.ethz.ch (D.M.); thomas.attin@zzm.uzh.ch (T.A.); tobias.tauboeck@zzm.uzh.ch (T.T.T.); 2 Department of Endodontics and Restorative Dentistry, School of Dental Medicine, University of Zagreb, 10000 Zagreb, Croatia; mpar@sfzg.hr; 3 Institute for Chemical and Bioengineering, Department of Chemistry and Applied Biosciences, ETH Zurich, 8093 Zurich, Switzerland; 4 Statistical Services, Center for Dental Medicine, University of Zurich, 8032 Zurich, Switzerland; daniel.wiedemeier@zzm.uzh.ch

**Keywords:** nanosized bioactive glass, dental adhesives, etch-and-rinse, self-etch, micro-tensile bond strength

## Abstract

This study investigated the short- and long-term effects of dental adhesives doped with nano-sized bioactive glass 45S5 (BAG) on the resin–dentin interfacial bond strength. Two etch-and-rinse adhesives (Adper Scotchbond Multi-Purpose (ASB) and Solobond Plus (SB)) and one self-etch adhesive (Clearfil SE Bond (CF)) were doped with different concentrations of BAG (5, 10, and 20 wt%). The unmodified (0 wt% BAG) commercial adhesives served as control groups. Dentin of 120 molars (n = 10 per group) was treated with the different adhesives, followed by buildups with a conventional composite restorative material. From each tooth, 14 sticks were prepared for micro-tensile bond strength (µTBS) testing. The sticks were stored in simulated body fluid at 37 °C and tested after 24 h or six months for µTBS and failure mode. Data were analyzed using Kruskal–Wallis tests in combination with post-hoc Conover-tests and Wilcoxon signed-rank tests at a level of significance of α = 0.05. After 24 h and six months, both etch-and-rinse adhesives with a low BAG content (up to 10 wt% for ASB and 5 wt% for SB) showed similar µTBSs as their respective control groups (0 wt% BAG). CF showed a significant decrease in µTBS even after addition of 5 wt% BAG. At a high concentration of added BAG (20 wt%), all three adhesives showed a significant decrease in µTBS compared to the unmodified controls. The CF control group showed significantly lower µTBS after 6 months of storage than after 24 h. In contrast, the µTBS of all CF groups modified with BAG was unaffected by aging. In conclusion, the tested etch-and-rinse adhesives can be modified with up to 5 wt% (SB), or 10 wt% (ASB) of BAG without reducing their short- and long-term dentin bond strength. Moreover, the addition of nano-sized BAG may prevent long-term bond strength deterioration of a self-etch adhesive.

## 1. Introduction

For more than 60 years, resin composites have offered an increasing range of indications compared to previous nonadhesive restoration materials [1,2,3]. Since then, resin composites have become the most widely used restorative materials in dentistry [4]. However, maintaining a durable interface between the tooth tissue and the restorative material remains a challenge [5]. The loss of interfacial integrity can lead to marginal gaps with associated postoperative sensitivity, marginal discoloration, and formation of secondary caries [6]. In addition, major failures of adhesive bonding can lead to the loss of the restoration [7].

The loss of integrity of the hybrid layer can result from poor penetration of the adhesive into the demineralized collagen network and subsequent enzymatic degradation by matrix metalloproteases (MMPs) [8,9]. Exogenous inhibitors such as chlorhexidine may at least partially prevent degradation of the hybrid layer [10]. Furthermore, it was suggested that bioactive glass 45S5 (BAG) can also inhibit the degradation of the hybrid layer to a certain extent [11]. Including BAG in the pretreatment of dentin before placing adhesive restorations or its incorporation into the restorative materials is intended to improve their properties. This is achieved not only by preventing degradation of the hybrid layer [12], but also by achieving remineralization of the adjacent tooth structure [12,13] and antibacterial activity [14,15]. Air abrasion with BAG prior to application of the adhesive system [16] and rewetting of the dentin with BAG-modified solutions [15] are approaches for integrating BAG into adhesive restoration processes. To avoid an additional step in the adhesive pretreatment, the functionalization of adhesives with customized fillers is another possibility to optimize dentin hybridization and prevent bond strength degradation. By using an experimental adhesive containing calcium- and phosphate-releasing micro-fillers, stable bond strength values could be demonstrated over a storage time of six months, whereas the nonfunctionalized commercial adhesive showed a decrease in dentin bond strength with time [17]. Other studies have examined the therapeutic effects of experimental adhesives doped with remineralizing compounds and have suggested an improved adhesive interface, increased mechanical properties such as hardness and modulus of elasticity [18,19,20], and no negative influence on the degree of conversion [21]. Recent studies have attempted to integrate BAGs into commercial adhesive systems. They successfully demonstrated bioactive attributes such as the precipitation of calcium and phosphate [22,23] without diminishing the microhardness and degree of conversion [23].

Originally, microparticulate BAG powders used as experimental fillers in dental materials were produced from a melt [24]. More recent attempts included the synthesis of nanometric BAG by the so-called flame spray process [25]. Compared with their microparticulate counterparts, BAG nanoparticles have been found to possess enhanced ion-releasing properties and induce higher remineralization rates [26,27,28]. This, together with their sub-micron size, specifically qualifies these particles as bioactive additives for bonding agents. However, the effect of doping dental adhesives with nano-scaled BAG on the early and long-term dentin bonding performance has not been investigated thus far. Being able to successfully incorporate these BAG particles into commercial dental adhesives and obtain stable long-term bond strengths would mean that an adhesive could be developed that would possess all the beneficial properties of BAG. Most of the studies that found beneficial effects of incorporating BAG into adhesive systems were, however, performed using micro-sized BAG (particle size range from <10 to 30 µm) [12,19,20,29], and only one study investigated the effect of nano-sized BAG [30].

As the downsizing of BAG fillers in dental materials has the potential benefits of improved reactivity [31], the need for lower amounts of BAG, and thus more space in the adhesive for other reinforcing fillers, the present in vitro study aimed to investigate whether admixing nano-sized BAG in different concentrations (5, 10, and 20 wt%) into etch-and-rinse and self-etch adhesives would affect their micro-tensile bond strength to dentin compared to the respective unmodified commercial adhesives. The null hypothesis was that the incorporation of BAG into adhesive systems would not affect their short- and long-term dentin bond strength.

## 2. Materials and Methods

### 2.1. Material Preparation

Nano-sized particles of BAG (45 wt% SiO_2_, 24.5 wt% Na_2_O, 24.5 wt% CaO, and 6 wt% P_2_O_5_) were produced by flame spray synthesis [25]. For this purpose, corresponding metal precursors [32] were mixed and combusted in a flame reactor. The particles were collected on a filter placed above the flame, and they were sieved subsequently (125 µm) to obtain a fine powder. Particle size diameters were determined by scanning electron microscopy [33]. Thus, prepared particles had a size of 30–50 nm.

As described previously by Tauböck et al. [23], the BAG particles were admixed into the bonding resin of three commercial adhesive systems. Two of these adhesives were etch-and-rinse adhesives: Adper Scotchbond Multi-Purpose (ASB; 3M, St. Paul, MN, USA) and Solobond Plus (SB; VOCO GmbH, Cuxhaven, Germany). The third adhesive was the self-etch adhesive Clearfil SE Bond (CF; Kuraray Noritake Dental Inc., Osaka, Japan). Table 1 lists the adhesive systems used in this study and their compositional details as provided by the manufacturers.

The BAG particles were incorporated in different concentrations (5, 10, and 20 wt%) into the bonding resin by using an asymmetric centrifuge (Speedmixer DAC 150, Hauschild Engineering, Hamm, Germany) for 60 s at increasing speed, followed by 90 s at a maximum speed of 3500 rpm.

### 2.2. Specimen Preparation

For this in vitro study, 120 extracted noncarious human molars were selected and irreversibly anonymized. All teeth were by-products of regular dental treatment, and patients gave written informed consent prior to the use of their teeth for research purposes. Under these terms, the research was compiled with the use of anonymized biological material, and authorization of the project from the local ethics committee was not necessary, as it did not fall within the scope of the Human Research Act (BASEC-Nr. Req.-2019-00717).

After extraction, the teeth were cleaned and stored in tap water at 5 °C until use. In order to allow precise manipulation, the teeth were fixated on a scanning electron microscope carrier (Wenka, Karl Wenger SA, Courgenay, Switzerland) using a light-curable resin (LC Block-Out Resin, Ultradent Products Inc., South Jordan, UT, USA) and embedded in self-curing acrylic resin (Paladur, Heraeus Kulzer, Hanau, Germany), 2 mm below the level of the cementoenamel junction. The teeth were ground to half of the clinical crown with 180-grit silicon carbide paper (Buehler-Met II, Buehler, Lake Bluff, IL, USA) by using a polishing machine (Planopol-2, Struers, Ballerup, Denmark) at low speed (150 rpm) under constant water cooling, leaving only dentin in the central area. To ensure that no enamel remnants were left in the central part and that the pulp was not exposed, the teeth were examined under a stereomicroscope (Stemi 1000, Carl Zeiss, Feldbach, Switzerland). Subsequently, the teeth were randomly allocated into 12 groups (n = 10 per group). Three of these groups were treated with the unmodified (commercial) adhesive systems and, thus, represented control groups (0 wt% of BAG). The remaining nine groups were treated with adhesives that had been infiltrated with 5, 10, or 20 wt% of BAG.

### 2.3. Restoration

**Figure 1:**
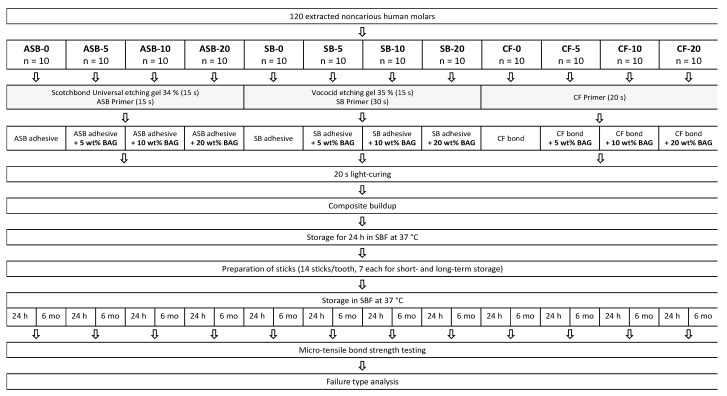
Experimental protocol. ASB: Adper Scotchbond Multi-Purpose; SB: Solobond Plus; CF: Clearfil SE Bond; BAG: bioactive glass 45S5; SBF: simulated body fluid.

The adhesive system application, composite buildups, and storage of the prepared specimens were performed as shown in Figure 1. Light-curing was performed for 20 s for all adhesive systems using an LED curing unit (Bluephase G2, Ivoclar Vivadent, Schaan, Liechtenstein; radiant exitance: 1200 mW/cm^2^) as close and perpendicular to the surface as possible.

For ASB, specimens were etched with 34% phosphoric acid (Scotchbond Universal Etchant, 3M, St. Paul, MN, USA; LOT: 5572623) for 15 s, rinsed with water for 15 s, and gently dried using an air syringe. Primer (Adper Scotchbond Multi-Purpose, 3M, St. Paul, MN, USA; LOT: NA37642) was rubbed in for 15 s and lightly air-blown for 5 s. The adhesive (Adper Scotchbond Multi-Purpose, 3M, St. Paul, MN, USA; LOT: NA44272) modified with either 0, 5, 10, or 20 wt% of BAG was applied according to the manufacturer’s instructions and light-cured.

Specimens pretreated with SB were etched with 35% phosphoric acid (Vococid, VOCO GmbH, Cuxhaven, Germany; LOT: 1923187) for 15 s and rinsed with water for 15 s. Primer (Solobond Plus Primer, VOCO GmbH, Cuxhaven, Germany; LOT: 1926411) was rubbed in for 30 s, and adhesive (Solobond Plus Adhesive, VOCO GmbH, Cuxhaven, Germany; LOT: 1915395), modified with either 0, 5, 10, or 20 wt% of BAG, was applied as per the manufacturer’s instructions. The adhesive was then light-cured for 20 s.

For specimens with the CF adhesive pretreatment, the primer (Clearfil SE Bond, Kuraray Noritake Dental Inc., Osaka, Japan; LOT: 3R0326) was rubbed in for 20 s and lightly air-blown for 5 s. Subsequently, the adhesive (Clearfil SE Bond, Kuraray Noritake Dental Inc., Osaka, Japan; LOT: 2T0543) modified with either 0, 5, 10, or 20 wt% of BAG was applied according to the manufacturer’s instructions and light-cured for 20 s.

After application of the adhesive systems, a transparent ring matrix (Lucifix Matrices, Kerr, Orange, CA, USA) was attached around the specimens. A nanofilled composite (Filtek Supreme XTE, 3M, St. Paul, MN, USA; shade A1, LOT: NA39148) was used to perform the buildups of 4–5 mm of height in three increments. Each increment was approximately 1.5 mm thick and light-cured for 20 s. Subsequently, the matrix was removed and the specimens were stored for 24 h in simulated body fluid (SBF) at 37 °C. SBF was prepared according to the formulation of Cho et al. [34]. The chemical compounds are listed in Table 2.

### 2.4. Micro-Tensile Bond Strength Test

The specimens were trimmed longitudinally in two directions with a water-cooled diamond saw (Accutom-50, Struers, Birmensdorf, Switzerland) and a diamond wheel (M0D10, Struers, Birmensdorf, Switzerland; diameter: 102 mm, thickness: 0.3 mm). The 14 most central, enamel-free sticks were marked and cut parallel to the surface of the tooth by a low-speed precision cutter (IsoMet, Buehler, Lake Bluff, IL, USA). It was ensured that only sticks without enamel were used by using a stereomicroscope (Stemi 1000, Carl Zeiss, Feldbach, Switzerland).

The 14 dentin–composite sticks of each tooth were stored in 5 mL of SBF at 37 °C. After 24 h, half of the obtained sticks (7 out of 14 sticks per tooth) were randomly chosen for micro-tensile bond strength (µTBS) testing. The other half was tested after six months of storage in SBF at 37 °C, which was changed weekly during this storage phase.

The micro-tensile bond strength test was performed using a universal testing machine according to the protocol of Armstrong et al. [35]. The dimensions of each stick were determined with a digital micrometer (406-250-30, Mitutoyo AG, Urdorf, Switzerland) to calculate the adhesive area. The mean adhesive surface area of the sticks was 0.907 ± 0.221 mm^2^. The sticks were then glued at both ends with cyanoacrylate glue (Renfert GmbH, Hilzingen, Germany) into µTBS jigs (Wenka, Karl Wenger SA, Courgenay, Switzerland), which were previously sandblasted with 110 µm aluminum oxide. The sticks were clamped in the universal testing machine (Zwick Roell Z010, Ulm, Germany), and a tensile force was applied until failure. The machine was operated at a speed of 1 mm/min using a load cell of 500 N. µTBS (MPa) was calculated from the load at failure (N) divided by the bonding area (mm^2^) of the respective stick.

### 2.5. Failure Analysis

Failure modes were analyzed under a stereomicroscope (Stemi 1000, Carl Zeiss, Feldbach, Switzerland) at 10× magnification. Adhesive failures, cohesive failures within the dentin, cohesive failures within the composite, and mixed failures were distinguished.

### 2.6. Statistical Analysis

Specimens that failed prior to µTBS testing were considered as pre-test failures (PTF) and were set to 0 MPa [35]. Due to the strongly nonnormal distribution of the dependent variable µTBS, a nonparametric approach was chosen. First, measurements of the individual sticks were aggregated per tooth by taking the median, leading to independent observations. Then, the effects of adhesive (ASB, SB, and CF) and BAG (0, 5, 10, and 20 wt%) on µTBS were separately investigated using Kruskal–Wallis omnibus tests followed by post-hoc Conover tests to compare pairwise differences between adhesives and BAG levels, respectively. Resulting *p*-values were corrected for multiple testing according to the Benjamini–Yekutieli procedure. The effect of storage time (24 h vs. six months) was investigated using Wilcoxon signed-rank tests. All statistical analyses and plots were computed with the statistical software R [36], including the packages PMCMR [37], rcompanion [38], and ggplot2 [39], at a pre-set level of significance of α = 0.05.

## 3. Results

### 3.1. Micro-Tensile Bond Strength

**Figure 2:**
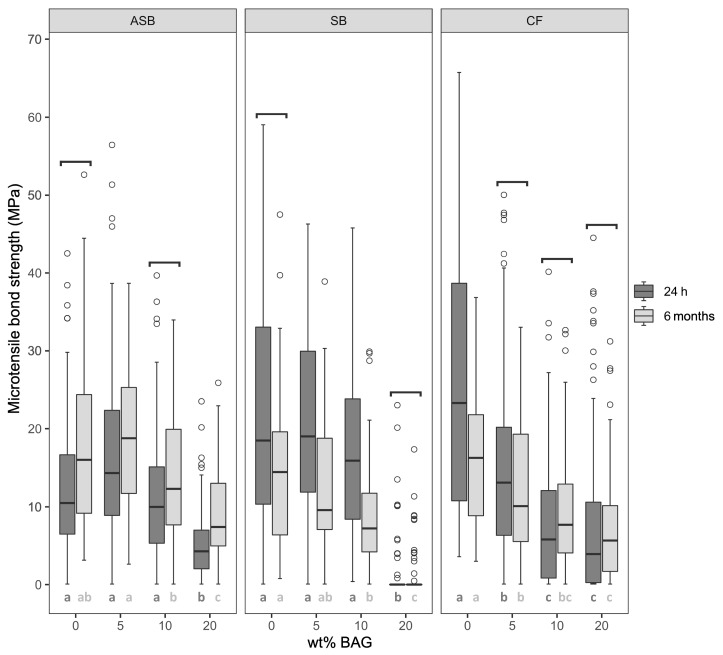
Micro-tensile bond strength (µTBS, in MPa) after 24 h and 6 months of storage in simulated body fluid (SBF). Boxplots show the medians (black lines) with 25 and 75% quartiles (boxes). The whiskers represent 1.5× interquartile range (IQR), or minima and maxima of the distribution if below 1.5× IQR. Outliers are shown as circles. The brackets above the box plots indicate no statistically significant differences (*p* ≥ 0.05) in µTBS between storage times. Statistically significant differences (*p* < 0.05) in µTBS within the adhesive groups on the level of bioactive glass content are marked with different letters. Dark gray letters refer to 24 h values, light gray letters refer to 6 months values. ASB: Adper Scotchbond Multi-Purpose; SB: Solobond Plus; CF: Clearfil SE Bond; BAG: bioactive glass 45S5.

Figure 2 presents the µTBS of all tested groups after 24 h and six months. For the etch-and-rinse adhesives (ASB and SB), no significant differences in µTBS were observed after 24 h between the control group and the groups modified with 5–10 wt% of BAG. Moreover, after 6 months of storage, ASB with 5 and 10 wt% BAG (ASB-5 and ASB-10), and SB with 5 wt% BAG (SB-5) revealed no significant µTBS differences compared to their respective control groups. For SB, a significant decline in µTBS was observed after 6 months of storage when modified with 10 wt% of BAG. After 24 h and six months, both etch-and-rinse adhesives modified with 20 wt% of BAG (ASB-20 and SB-20) showed significantly lower µTBS values compared to the control groups.

The control groups of both etch-and-rinse adhesives (ASB-0 and SB-0) showed no significant changes in their dentin bond strength between 24 h and six months. SB modified with 5–10 wt% of BAG (SB-5 and SB-10) showed a significant reduction in µTBS after 6 months, which was not observed when SB was modified with 20 wt% of BAG (SB-20). In contrast, for ASB modified with 5 and 20 wt% of BAG (ASB-5 and ASB-20), the bond strength significantly increased after the 6 months of storage compared to the results obtained after 24 h.

CF showed a significant decrease in µTBS compared to the control group for all BAG concentrations (5–20 wt%) after both 24 h and 6 months. Whereas the control group of the self-etch adhesive (CF-0) showed a significant decline in µTBS after 6 months of aging, the µTBS of CF modified with 5–20 wt% of BAG (CF-5, CF-10, and CF-20) remained unaffected by aging.

### 3.2. Failure Analysis

**Figure 3:**
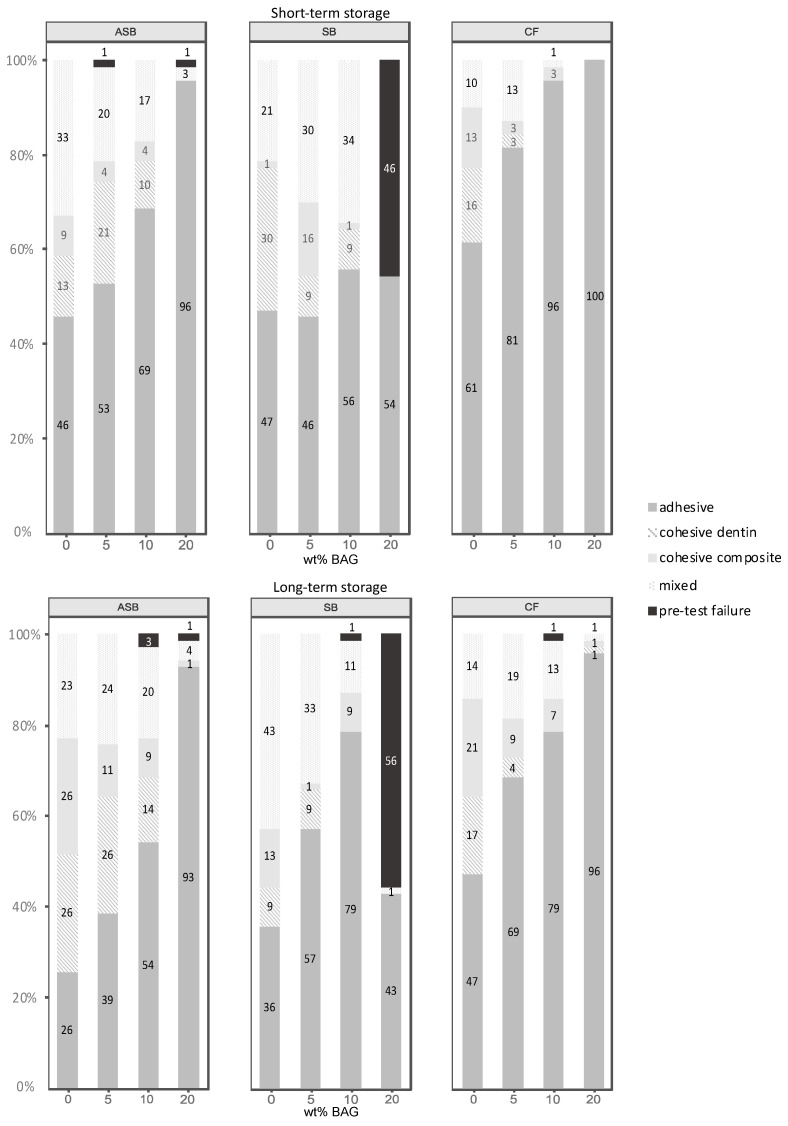
Distribution of failure modes in percentages for the short-term (24 h) and long-term (6 months) storage. ASB: Adper Scotchbond Multi-Purpose; SB: Solobond Plus; CF: Clearfil SE Bond.

Figure 3 shows the frequency distribution of failure modes of all groups.

Both after 24 h and six months of storage, the percentage of adhesively failed sticks increased with higher concentrations of BAG. The percentage of cohesive or mixed failures was highest in the control groups (ASB-0, SB-0, and CF-0) and decreased with the concentration of BAG. An exception to this pattern was observed when SB was modified with 20 wt% of BAG (SB-20), which showed a high incidence of PTFs. In this group, 46% were identified as PTFs after 24 h, whereas after six months, more than half of the sticks (56%) failed before testing. In contrast, all ASB and CF groups, including those with 20 wt% of BAG, showed nearly no PTFs.

## 4. Discussion

The tested null hypothesis was rejected as the incorporation of BAG into the adhesive systems affected both the short-and long-term dentin bond strength. Whereas for the etch-and-rinse adhesives (ASB and SB), no significant effect on µTBS was identified after 24 h for 5 and 10 wt% of added BAG, as well as after six months for 5 wt% of added BAG, the self-etch adhesive (CF) showed a significant reduction in dentin bond strength even at low concentrations of BAG (5 wt%), both after 24 h and six months.

Designing completely new experimental dental adhesives is complex, as factors such as pH and hydrophilicity considerably influence material behaviors [18,39,40,41]. Therefore, commercial etch-and-rinse (ASB and SB) and self-etch (CF) adhesives were used in the present study and modified with nano-sized BAG. The resin component (“second bottle”) of the adhesive systems showed a higher pH as the primer component (“first bottle”) and was therefore used for admixing the BAG fillers [42,43], as indicated in Table 1. Adding alkaline BAG into an acidic solution might result in an acid–base reaction, which would impair both the infiltrating capability of the primer and bioactive properties of the filler [44].

The specimens were stored in SBF to provide ions that could react with the components of the BAG. In the presence of SBF, a release of Na^+^ and, additionally, Ca^2+^, PO_4_^3-^, and Si^4+^ occurs from the BAG surface. A silica-rich layer is formed on the glass body, which acts as a nucleation site for the formation of calcium phosphate precipitates [45]. Together with the calcium and phosphate ions from the surrounding SBF, calcium phosphate crystallization is further enabled [46]. These reactions have previously been shown to remineralize demineralized tooth structure [23,47]. Over the 6 months storage period, the immersion medium was changed weekly to prevent colonization of bacteria. Besides, a constant supply of fresh fluid was ensured to simulate clinical conditions as close as possible. The 6 months storage period was intended to give the components of the BAG and the ions from the SBF enough time to react with each other and form calcium phosphate precipitates. Degradation of the collagen fibers by MMPs is also usually not detected until after 3 to 6 months [48].

For ASB functionalized with 5 and 20 wt% of BAG (ASB-5 and ASB-20), a significant increase in µTBS was observed after six months. The improvement of bond strength over time might be explained by the relaxation of residual contraction stresses due to storage in an aqueous environment. Although the adhesives were applied in a thin layer and light-cured under low constraint, the polymerization of multifunctional methacrylates inevitably leads to a buildup of contraction stresses [49,50]. These stresses render adhesive interfaces less resistant to external forces generated during µTBS testing. As the specimens age and absorb water, residual stresses are partially relieved [51], which might have increased µTBS values. In addition, the post-cure increase in the degree of conversion (DC) might be another explanation for the observed bond strength increase over the 6 months aging period [52]. Although the post-cure DC increase occurring beyond 24 h may be modest in absolute values, it considerably contributes to crosslinking density, thereby strengthening the polymeric network [53], which could have reflected as an improvement in µTBS.

For the etch-and-rinse adhesives SB and ASB, the addition of low concentrations of BAG (up to 5 and 10 wt%, respectively) had no significant effect on µTBS compared to the unmodified control groups. In contrast, the µTBS of the self-etch adhesive (CF) was significantly affected for all concentrations of added BAG (5–20 wt%). This behavior can be discussed in terms of the pH of the resin component (“second bottle”) to which BAG was admixed. The pH of CF is in a highly acidic range (2.3–2.4), SB has a less acidic pH (4.6), whereas the pH of ASB is in a “neutral” range (exact value not disclosed by the manufacturer, Table 1). In this regard, the less acidic adhesives seem to be more suitable for incorporating the alkaline BAG. This rationale also supports the concept of admixing BAG into the less acidic resin component instead of the more acidic primer component of the adhesive systems.

In the unmodified control groups of the etch-and-rinse adhesives (ASB-0 and SB-0), the 6 months of storage did not affect dentin bond strength. Etching with phosphoric acid might not only cause a release of acid-activated MMPs, but also denatures those enzymes [54,55] and might thereby prevent the degradation of exposed collagen [56]. Furthermore, the lack of acidic monomers in these adhesive systems might prevent further demineralization of the hybrid layer caused by remaining unpolymerized monomers after curing. In contrast, a significant deterioration of µTBS was observed in the unmodified control group of the self-etch adhesive (CF-0) after the 6 months of storage. MMPs are probably activated by the low pH of self-etch adhesives, but not denatured as they are by phosphoric acid [54]. Consequently, the activated MMPs may have led to reduced integrity of the hybrid layer and impaired strength of the resin–dentin interface [11,57]. On the other hand, the experimental CF groups with 5–20 wt% of added BAG showed stable µTBS over six months of artificial aging. Therefore, the addition of alkaline BAG might have neutralized the acidity within the hybrid layer produced by the application of CF, resulting in less activation of MMPs [13,15]. Moreover, remineralization through ions such as Ca^2+^, PO_4_^3−^, or Si^4+^ released from BAG might have fossilized endogenous proteases [58,59], resulting in stable µTBS values after the 6 months of storage. Additionally, Wang et al. [60] supposed that self-etch adhesives do not fully polymerize, due to residual water in dentinal tubules. Unpolymerized acidic monomers can be expected to continue demineralizing dentinal tissue, thereby reducing the structural integrity of the hybrid layer and diminishing adhesive bond strength with time [60]. It may be hypothesized that BAG particles incorporated into CF have not only prevented MMPs from being activated but also neutralized remaining acidic monomers to a degree that they could no longer manifest their acidic effect within the hybrid layer.

All three adhesive systems modified with 20 wt% of BAG showed significantly reduced µTBS compared to their respective unmodified (commercial) control groups. This might be explained by the fact that the viscosity of the adhesives was increased by the addition of BAG, which impeded penetration of the adhesive into the dentinal tubules, thus leading to reduced dentin bond strength [61].

An increased ratio of adhesive failures relative to other failure modes was observed in all investigated adhesive systems with increasing BAG concentrations. The evidence of a weakened adhesive interface was particularly pronounced in SB modified with 20 wt% of BAG (SB-20), in which 46–56% of the sticks failed before testing. Already during the process of incorporating BAG into the acetone-based adhesive resin of SB, a considerable increase in viscosity was observed, which made the handling more difficult. The high volatility of acetone as a solvent in this adhesive or an incomplete dispersion of BAG could have had a negative influence on the viscosity of the adhesive [22].

In addition to reduced penetration of the more viscous BAG-modified adhesives, a lower DC due to the higher filler content could be a further cause for reduced µTBS of experimental adhesives. Some studies have shown a negative effect of higher BAG concentrations on the DC of experimental composites [31,62,63,64]. Sufficient polymerization of the adhesive could be inhibited at high BAG concentrations, which however seems to depend on the composition of the adhesives [64,65]. For a more detailed understanding of the chemomechanical properties of BAG-modified adhesives, further investigations are required.

Finally, it has to be considered that the impact of admixing BAG into a dentin adhesive was shown for distinguished and deliberately chosen products. Therefore, the results could not be easily transferred to other adhesives with different compounds, as compared to the tested ones.

## 5. Conclusions

Within the limitations of this in vitro study, the following can be concluded:

1. The etch-and-rinse adhesives could be functionalized with 5 wt% (Solobond Plus) or up to 10 wt% (Adper Scotchbond Multi-Purpose) of nano-sized bioactive glass with no negative effect on their dentin bond strength.
2. Although addition of bioactive glass to the self-etch adhesive (Clearfil SE Bond) significantly diminished its performance for all bioactive glass concentrations, a beneficial effect was identified in terms of maintaining stable dentin bond strength over the 6 months aging period.

The capability to maintain bond strength when functionalized with nano-sized bioactive glass and the potential for improved longevity of the bonded interface makes the bioactive glass-modified adhesive systems promising candidates for further investigations.

## Figures and Tables

**Table 1:** Composition of the adhesive systems used in this study according to manufacturers’ specifications.

Product	Manufacturer	Type	wt%	Composition	pH	LOT
Scotchbond Universal Etchant	3M, St. Paul, MN, USA			**Etchant**:	<1	5572623
			55–65	Water		
			30–40	Phosphoric acid		
			5–10	Silica		
			1–5	Polyglycol		
			<2	Aluminum oxide		
Adper Scotchbond Multi-Purpose Adhesive (ASB)	3M, St. Paul, MN, USA	3-step etch-and-rinse		**Primer**:	2.9–4.0	NA37642
			40–50	Water		
			35–45	HEMA ^1^		
			10–20	Copolymer of itaconic and acrylic acid		
				**Adhesive**:	neutral	NA44272
			60–70	Bis-GMA ^2^		
			30–40	HEMA		
			<0.5	Triphenylatimone		
			<0.2	Triphenylphosphine		
			<0.05	Hydroquinone		
Vococid^®^	VOCO GmbH, Cuxhaven, Germany			**Etchant**:	0.8	1923187
			25–50	Phosphoric acid		
Solobond Plus (SB)	VOCO GmbH, Cuxhaven, Germany	3-step etch-and-rinse		**Primer**:	2.5	1926411
			10–25	HEMA		
			10–25	Acetone		
			10–25	Hydroxypropyl methacrylate		
			≤2.5	Catalyst		
				**Adhesive**:	4.6	1915395
			50–100	Acetone		
			10–25	Bis-GMA		
			10–25	TEGDMA ^3^		
			5–10	HEMA		
			≤2.5	Catalyst		
Clearfil SE Bond (CF)	Kuraray Noritake Dental Inc., Osaka, Japan	2-step self-etch	20–40	**Primer**: HEMA 10-MDP ^4^, camphorquinone, hydrophilic dimethacrylate	2.0	3R0326
			25–45 20–40	**Bonding**: Bis-GMA HEMA 10-MDP, aliphatic dimethylacrylate, dl-camphorquinone, accelerator, water, colorants	2.3–2.4	2T0543

^1^ HEMA: 2-hydroxylethyl methacrylate; ^2^ Bis-GMA: bisphenol-A-glycidyl-dimethacrylate; ^3^ TEGDMA: triethylene glycol dimethacrylate; ^4^ 10-MDP: 10-methacryloyloxydecyl dihydrogen phosphate.

**Table 2:** Chemical compounds of simulated body fluid (SBF) used.

Compounds	Amount in 1000 mL
NaCl	7.995 g
(HOCH_2_)_3_CNH_2_	6.055 g
CaCl_2_	0.368 g
NaHCO_3_	0.353 g
MgCl_2_	0.305 g
KCl	0.224 g
K_2_HOP_4_	0.174 g
Na_2_SO_4_	0.071 g
1.0 M HCl	40 mL

## Data Availability

The data presented in this study are available on request from the corresponding author.
